# EU enlargements, Brexit and value-added trade: A structural gravity approach

**DOI:** 10.1371/journal.pone.0299738

**Published:** 2025-04-16

**Authors:** Jan Hagemejer, Jakub Mućk

**Affiliations:** 1 University of Warsaw, Warsaw, Poland,; 2 CASE Center for Social and Economic Research, Warsaw, Poland,; 3 SGH Warsaw School of Economics, Warsaw, Poland; MIT: Massachusetts Institute of Technology, UNITED STATES OF AMERICA

## Abstract

We revisit the topic of trade effects of past EU enlargements. We employ a structural gravity model to flows of domestic value added embedded in international trade and absorbed in the foreign final demand for the period of 1995–2018. Our model takes into account the current state of the art in estimation of gravity models: PPML estimation and multi-dimensional fixed effects. We base our estimations on the TiVA dataset and provide aggregate and sectoral estimates. Our results show stable estimates of EU enlargement on value-added trade of the order of 13.9% depending on the specification. These results are also robust to a choice of an alternative dataset (WIOD database). The advantage of our approach is the direct calculation of the trade impact on the GDP level. Based on these estimates we also provide quantitative insights into the effects of Brexit which varies depending on the country-specific exposure to the value added trade with the UK.

## 1 Introduction

In 2020 the United Kingdom (UK) left the European Union (EU). The Trade and Cooperation Agreement between the two parties signed on the 24^th^ of December 2020 and in effect from 2021 has changed the trade relations between UK and the EU from a deep economic integration arrangement to a free trade area. This means that tariff free trade is maintained, but both parties can freely shape their trade policy towards the third countries and, more importantly, technical regulations and standards are no longer harmonised or mutually recognised, border controls are reintroduced, and the non-tariff barriers are expected to increase. This arrangement is very similar to the one enjoyed by New Member States of the EU (NMS thereafter, this group comprises of: Bulgaria, Croatia, Cyprus, Czech Republic, Estonia, Hungary, Latvia, Lithuania, Malta, Poland, Romania, Slovakia, and Slovenia.) in their respective pre-accession periods. This similarity is the main motivation for the approach taken in this paper.

In this paper, we revisit the trade effects of European integration. More specifically, we focus on the effects of the 2004, 2007 and 2013 EU enlargements on trade flows. However, unlike the bulk of existing literature, we make use of the methodological developments on the topic of global value chains (see, e.g., [1]) and base our estimates on the value-added flows. This is enabled by the availability of data, i.e., the TIVA database that spans the period of 1995–2018, covering data points before and after accession. We follow the state-of-the art of gravity model estimation literature (see, e.g., [2–4]) by employing a structural gravity model for theory-consistent estimation. Our estimations are based on aggregate as well as sectoral bilateral value-added flows. As the post-Brexit data on value-added trade flows are yet unavailable, it is too early to provide direct estimates of the trade effects of Brexit. In our identification strategy, we use the fact that Brexit is indeed a reversal of the accession process and therefore the robust estimates of the trade effects of EU enlargement for multiple countries over more than a decade serve as a basis for the projection of the effects of Brexit.

The value-added approach has important advantages as opposed to standard estimations based on gross trade. First, trade in value added is consistent with national accounts and therefore these estimates are easily converted to contributions to gross domestic product (GDP) growth and therefore provide a direct estimate of the macro effects without the need to impose a theoretical framework to compute these effects (such as a general equilibrium model). Second, the dataset used covers both trade in goods and services consistently, taking into account both the direct exports of value added as well as exported value added of non-exporting manufacturing and service sectors *embedded* into gross exports of exporting sectors. Third, as the EU countries are interlinked in production chains, the export-oriented output has been increasingly import-intensive which makes the link between gross exports and GDP cumbersome (i.e., export-led growth) – the value-added trade approach solves this problem by accounting for import intensity of exports.

This study contributes to at least two strands of literature: the ample strand of literature on the effects of preferential trading agreements and European integration [5–8], based on the gravity model estimations as well as the literature analysing the value-added flows in the context of European (see, e.g., [9,10] and [11]). The approach to gravity estimations based on value-added flows, joint treatment of manufacturing and service sectors, and the identification of the effects Brexit using the obtained gravity estimates are original compared to the available literature.

This paper is structured as follows. Section 2 reviews the relevant literature. Section 3 contains the methodology and data description. Section 4 presents the baseline results of gravity estimation. Section 5 provides more detailed insights into the structure of gains from EU enlargement. Section 6 provides estimates of the likely effects of Brexit. Section 7 covers the additional robustness checks. The last section concludes.

## 2 Review of literature

The topic of European integration had its peaks of popularity in the economic literature on several occasions. One of them was the completion of the internal market through the Single Market Program of 1992 announced in the 1985 Commission of European Communities White Paper [12] and implemented in the Single European Act of 1992. This large structural change introducing a single market guaranteeing the free movement of goods, capital, services, and people spurred the first wave of ex-ante studies. These start with the so-called Chechini report [13] providing a comprehensive sectoral and macroeconomic study of the impact of the removal of remaining trade barriers as well as such notable works as [14–17]. These early works emphasize the effects of increased competition on the better allocation of resources and in turn, welfare as well as the removal of non-tariff barriers together with EU standardization policy (harmonization of product regulations) and were mainly based on simulation models (either partial or general equilibrium) that were gaining popularity at that time.

The second wave of *ex-ante* studies started with the prospective EU enlargement of 2004 and 2007. The process started in the early 1990s when the Central and Eastern European (CEE) countries signed a series of EU Association Agreements and committed to comprehensive reforms increasing transparency and competition, improving market and democratic institutions, as well as adjusting domestic laws towards the European standards. This also involved gradual trade liberalization: the CEE formed several free trading agreements between themselves: e.g., CEFTA (in 1992, originally between Czech Republic, Slovakia, Poland, and Hungary, later extended to more countries, BAFTA (between Baltic countries), and other bilateral agreements (see [18,19]). However, the EU Association Agreements also triggered bilateral trade liberalization with the EU; by 2000 most tariffs (except for sensitive agricultural products) were already gone in bilateral trade between the EU and the first wave of prospective members. A large part of the effects of tariff liberalization and the reforms has materialized before the actual EU accession, with the accession itself being similar in scope to the introduction of the 1992 program: i.e., removal of remaining non-tariff barriers and unrestricted access to the single market (further strengthened in services by the Services Directive and through the delayed unrestricted movement of the labour force). Early macroeconomic evaluations of the welfare effects of accession used a similar methodology to those employed for the original 1992 program. The notable works include, among others: [16,20] and [21] showing significant macroeconomic impact of EU accession.

Over two decades after the 2004 EU enlargement, the topic has emerged together with centrifugal forces within the EU and the increased popularity of economic nationalism across Europe culminating in the 2021 Brexit Agreement and the actual departure of the United Kingdom from the EU. The emergence of data sets spanning several decades has enabled scholars to look at the long-term effects of EU integration. There have also been critical developments in empirical methodologies. These include the developments in the gravity model framework, in particular, the theory-consistent methods of estimation (for an overview see [22]) allowing for unbiased estimates of the effects of trade agreements. A notable application of this methodology is the ex-post estimate of the costs of non-Europe [23] showing large but differentiated welfare gains of EU integration. Considerable trade effects of EU integration are obtained in [8] using a structural gravity model. These and other works are, however, based on gross trade statistics.

Another important contribution is the development of the synthetic counterfactual method that relies on building a hypothetical reference scenario ([24]) and its application to the EU membership [25] showing significant divergence between the integration and non-integration scenarios of the pre-2004 members of the EU. Similar effects for 2004 and 2007 enlargements are obtained in [26].

Finally, there have been several attempts to quantify the effects of Brexit. They include both computable models of different flavours (see, e.g., [27]), macro models ([28,29]) and structural gravity estimates ([30–32]). The gravity models presented in those studies are estimated using gross trade statistics and serve as the basis for calibration of general equilibrium models that in turn provide macroeconomic effects of Brexit. Our approach, due to direct application to value-added trade allows for simpler identification of the effect of Brexit. In the last section, we compare the obtained results to selected studies.

## 3 Methodology and data

The theoretical equation underlying our estimating equation is the model by Anderson and van Wincoop ([2], see also [4]):


$$T_{ij}=\frac{Y_{i}E_{j}}{Y}{\left(\frac{t_{ij}}{{\text{Π}}_{i}P_{j}}\right)}^{1-\sigma },$$ (1)

where $T_{ij}$ is the trade flow between exporter *i* and importer *j*, $Y_{i}$ is the income of the exporting country, $E_{j}$ is the expenditure of the importing country, Y is world income, $t_{ij}$ is the bilateral cost of trade between within each i-j country pair, ${\text{Π}}_{i}$ and $P_{j}$ are the multilateral resistance terms for the exporting (outward) and importing (inward) country respectively and *σ* is the elasticity of substitution between varieties traded coming from different countries.

The empirical approach is the gravity model in the form proposed by Silva and Tenreyro [3], i.e., the Poisson pseudo maximum likelihood model (PPML). Using PPML solves the common selection problem in gravity estimation, i.e., the zero trade flows between selected pairs of countries. Moreover, thanks to the multiplicative form (instead of the original linearized gravity equation), helps with the treatment of heteroskedasticity in the trade data. The empirical model takes the following form:


$$VA_{ijt}=\exp\left(\beta_{0}+\delta_{ij}EU_{ijt}+\beta rta_{ijt}+\gamma wto_{ijt}+\lambda X_{ijt})+u_{it}+u_{jt}+u_{ij}\right)\times \varepsilon_{ijt},$$ (2)

where $VA_{ijt}$ is the value added produced in the *i*-th country and consumed in goods and services in the *j*-th economy at time *t,* the our measure of trade $T_{ij}$. The key variable of interest, i.e., $EU_{ijt}$ is an indicator variable which takes one if two trading economies belong to the European Union at time *t*. Importantly, the $EU_{ijt}$ is time-varying as the sample will cover the period in which new countries joined the European Union and therefore the estimate on this dummy variable would correspond to the effect of EU enlargement.

It is important to note, that our specification is purely focused on within variation, as we include the pair-specific fixed effects $u_{ij}$ that help to solve several problems related to endogeneity (see, e.g., [33]). They are perfectly collinear with all the time-invariant bilateral variables, including the standard gravity ones (e.g., distance, colonial ties, contiguity) and hence these variables, absorbed by fixed effects, are absent from the regressions. Therefore, the effects of bilateral trade costs in the theoretical equation $t_{ij}$ are absorbed by the pair-specific fixed effects as well as bilateral time-varying variables: participation in the World Trade Organisation (WTO, denoted as $wto_{ijt}$) and preferential regional trade agreements $rta_{ijt}$ and other bilateral time-varying variables (included in later versions of the model).

We also include time-varying fixed effects $u_{it}$ and $u_{jt}$ that are used to account for unobserved outward and inward multilateral resistance terms ${\text{Π}}_{i}$ and $P_{j}$. These fixed effects also capture the exporter output $Y_{i}$ and the importer demand $E_{j}$ and any unobserved factors both on the exporter and exporter side. Such an approach to gravity estimation provides a high level of robustness but only allows for time-varying bilateral control variables and hence, it is not possible to obtain estimates for the pre-2004 EU membership status effects as it does not change within the sample size. It is also important to note that the changes to economic activity that result from EU integration and the reforms undertaken by the prospective member states are already captured by the time-varying fixed effects and therefore the EU dummy is purely the effect on trade that is resulting from increased trade in the EU.

A key difference in our empirical strategy from the existing literature is the outcome variable. Instead of explaining the gross exports, we use the exports of value added. Specifically, we use the measure of value added that is generated domestically in the country of origin and ultimately absorbed in the destination country. This includes the value added in sectors that export directly to the destination countries, the value added which is produced in domestic non-exporting sectors (e.g., some service sectors) and sold to manufacturing sectors and then embedded in such sectors exports, as well as value added that is first embedded in intermediate goods sold to a third country in the form of intermediate goods and then ultimately consumed in the form of final goods in the destination country. Therefore, the effects of EU enlargement we observe capture the effects of trade that occurs within the European Union when the final consumer (in the form of private, government or investment demand) is in the EU and at least some part of the production process is in the EU. Our $EU_{ijt}$ dummy does not capture the effects of increased trade in intermediate goods that ends up being exported outside the EU.

In further regressions, we assume that the effect of EU enlargement in equation (1) is heterogeneous. Although this strategy is quite common, in section 4 we additionally investigate possible heterogeneity in this effect by including interaction variables with both the importer and exporter country. Moreover, we also are interested how these integration effects change with distance between the exporter and importer as well as the size of the former and the latter. We capture those effects by employing appropriate interactions. Moreover, we also take into account that our data comes in sectoral aggregation (NACE 2-digit for some sectors, in some cases aggregates of two or three sectors) and we use these sectoral data to rerun our empirical specification. Through the use of interactions, we distil the effects of economic integration on production sectors.

Our principal database is the OECD TiVA (trade in value added) database [34]. It offers detailed information about industry linkages between 45 sectors in 66 countries over time span ranging from 1995 to 2018. At the time of preparation of the article, the newer edition of TiVA 2023 ranging between 1995 and 2020 according to its authors is very preliminary, in particular for the years after 2018, hence the choice of the well-established dataset, TiVA 2021. This data is complemented with the standard gravity variables, i.e., distances, data on WTO membership, data on participation in preferential trading agreements including the EU membership. The source of these data is CEPII gravity dataset [35].

S1 Table in the supporting information shows all the variables used together with their sources, S2 Table shows the summary statistics and table S3 Table – pairwise correlations.

## 4 Baseline results

In this section, we provide baseline estimates of the effects of EU enlargements on the exported value added. Table 1 presents the baseline results. Columns (1)-(3) contains estimates for the bilateral flows in value added between countries. It is straightforward to observe that integration within European Union boosts the exported value added by around 13.9% (the exact effect is obtained by calculating exp(0.130)). Importantly, this number remains similar even if control variables are included in the regression. Other included variables also turn out to be positive and significant, i.e., WTO membership is associated with a boost in exported value added by around 7% while membership in the preferential trading agreements – 2.4%.

**Table 1:** Baseline estimated effect of European integration on exported value added.

	(1)	(2)	(3)	(4)	(5)	(6)
	Aggregate data			Sectoral data		
$EU_{ijt}$	0.130***	0.130***	0.130***	0.132***	0.132***	0.131***
	(0.0102)	(0.0102)	(0.0102)	(0.00344)	(0.00344)	(0.00344)
$wto_{ijt}$		0.0670*	0.0668*		0.0540***	0.0539***
		(0.0364)	(0.0364)		(0.0132)	(0.0132)
$rta_{ijt}$			0.0239***			0.0141***
			(0.00741)			(0.00267)
*N*	102,960	102,960	102,960	4,497,147	4,497,147	4,497,147
$R^{2}$	0.996	0.996	0.996	0.994	0.994	0.994

Note: ***, ** and * denote the rejection of null about parameters’ insignificance at 1%, 5% and 10% significance level, respectively. The expressions in round brackets stand for robust standard errors. Estimations based on the TiVA dataset for the period of 1995–2018.

In the next step, we consider more detailed data to estimate underlying parameters. The next three columns, (4)-(6), summarize estimation results for data in which the exported value added is additionally assigned to the industry. Using more granular data allows us to cross-check whether key estimates are robust to potential heterogeneity between industries. However, the differences between these estimates are negligible. This suggests that our key estimate implying the 13.9% premium for European integration is quite robust.

## 5 Heterogeneity in the effect of EU enlargements

We account for further heterogeneity in the effect of EU enlargement. The baseline results presented in section 3 are based on the assumption that this effect is homogeneous. However, it could be different across countries as well as across industries.

To account for such heterogeneity the key variable $EU_{ijt}$ is interacted with relevant country-specific or sector-specific dummy variables. Table 2 presents results in which the effect is different for exporters (columns (1) and (3)) or importers (columns (2) and (4)) while the specific estimates are plotted in S1 Fig. in the supporting information.

**Table 2:** Estimates of heterogeneity in the effect of European integration.

	(1)	(2)	(3)	(4)
	Aggregate data		Sectoral data	
$wto_{ijt}$	0.0670*	0.0616*	0.0443***	0.00475
	(0.0364)	(0.0364)	(0.0132)	(0.0134)
$rta_{ijt}$	0.0245***	0.0228***	0.0145***	0.0117***
	(0.00743)	(0.00743)	(0.00267)	(0.00267)
average $EU_{ijt}$	0.145***	0.196***	0.149***	0.204***
	(0.00979)	(0.0109)	(0.00344)	(0.00378)
HO: homogeneous *δ*	[0.000]	[0.000]	[0.000]	[0.000]
*N*	102,960	102,960	4,497,147	4,497,147
$R^{2}$	0.997	0.997	0.994	0.994

Note: ***, ** and * denote the rejection of null about parameters’ insignificance at 1%, 5% and 10% significance level, respectively. The expressions in round brackets stand for robust standard errors while the numbers in the squared brackets denote the probability values for the respective null. Estimations based on the TiVA dataset for the period of 1995–2018.

At the aggregate level, the estimated premium from EU enlargement is slightly above the baseline results. Furthermore, the formal statistical test allows to reject the null of homogeneity in *δ*. This is also supported by visual inspection of the estimated effect which in some cases could be negative and varies from −*.*2 to*.*5.

To document possible regularities in the premium for EU enlargement we extend our structural gravity model by the interaction of the $EU_{ijt}$ with fundamental gravity variables, i.e., distance (in logs, denoted by $logdist_{ij}$) and size of trading economies measured by their GDP ($GDP_{i}^{t}$ and $GDP_{j}^{t}$, denoting the GDP for exporter and importer, respectively.) With such choice, we investigate how European integration is able to go against the usual gravity-based pattern of trade, i.e., decrease the role of distance in shaping the costs of trade, as well as lower the role of the country size in driving the export demand and supply.

Table 3 summarizes estimates of the extended structural gravity model. In fact, all interactions are statistically significant while the estimates on control variables are very close to previous results. Interestingly, these results show that European integration mitigates the effect of standard (gravity) trade forces. For instance, the estimate on the logged distance is positive which means that trade cooperation within the EU allows to access more distant markets by reducing the trade costs (i.e., reduces the so-called iceberg effect). It might also be related to a high degree of vertical specialization. Since the production process became highly fragmented, more intermediates are exported in order to satisfy final demand in markets more geographically distant.

**Table 3:** Estimates of heterogeneity in the effect of European integration.

	(1)	(2)	(3)	(4)
	Aggregate data		Sectoral data	
$wto_{ijt}$	0.0551	0.0535	0.0533***	0.0507***
	(0.0365)	(0.0364)	(0.0132)	(0.0129)
$rta_{ijt}$	0.0256***	0.0256***	0.0153***	0.0161***
	(0.00741)	(0.00740)	(0.00267)	(0.00268)
$EU_{ijt}$	1.541***	1.542***	-0.648***	0.896***
	(0.165)	(0.164)	(0.0185)	(0.0571)
$EU_{ijt}\times logdist_{ij}$	0.157***	0.145***	0.113***	0.146***
	(0.00838)	(0.00820)	(0.00262)	(0.00281)
$EU_{ijt}\times logGDP_{i}^{t}$		-0.0771***		-0.0574***
		(0.00542)		(0.00187)
$EU_{ijt}\times logGDP_{j}^{t}$		-0.0495***		-0.0324***
		(0.00527)		(0.00188)
*N*	102,960	102,960	4,497,147	4,497,147
$R^{2}$	0.996	0.996	0.994	0.994

Note: ***, ** and * denote the rejection of null about parameters’ insignificance at 1%, 5% and 10% significance level, respectively. The expressions in round brackets stand for robust standard errors. Estimations based on the TiVA dataset for the period of 1995–2018.

The same applies the role of the economy’s size. Negative estimates on the exporter’s and importer’s GDP translate into the case in which smaller and less advanced economies benefit to a larger extent from European integration. This relationship also confirms the role of regional value chains where less developed economies act as a manufacturing backbone to more developed ones (e.g., Poland, Slovakia and the Czech Republic producing for German manufacturing). Viewed from a theoretical point of view, one might relate this to fixed costs of exporting. For firms in smaller and less advanced economies, it is easier to specialize only in production of selected intermediates or final goods to improve the market share by minimizing the magnitude of fixed costs relative to aggregate production value.

On the demand side, the negative coefficient can be explained by the fact that operating in each destination is also associated with some non-negligible fixed costs (e.g., adjustment of products to local preferences) which could be disproportional to the size of the market. Therefore, by cooperating within regional value chains this cost may be distributed over firms in different production stages, making sales on smaller markets more profitable.

Finally, we also investigate heterogeneity at the industry level. To do so, we rerun the panel structural gravity model for each industry separately. This choice stems from the fact that both the effect from European integration and, in a more general setting, from trade liberalization, which is captured by control variables, could be product- or industry-specific.

**Fig 1:**
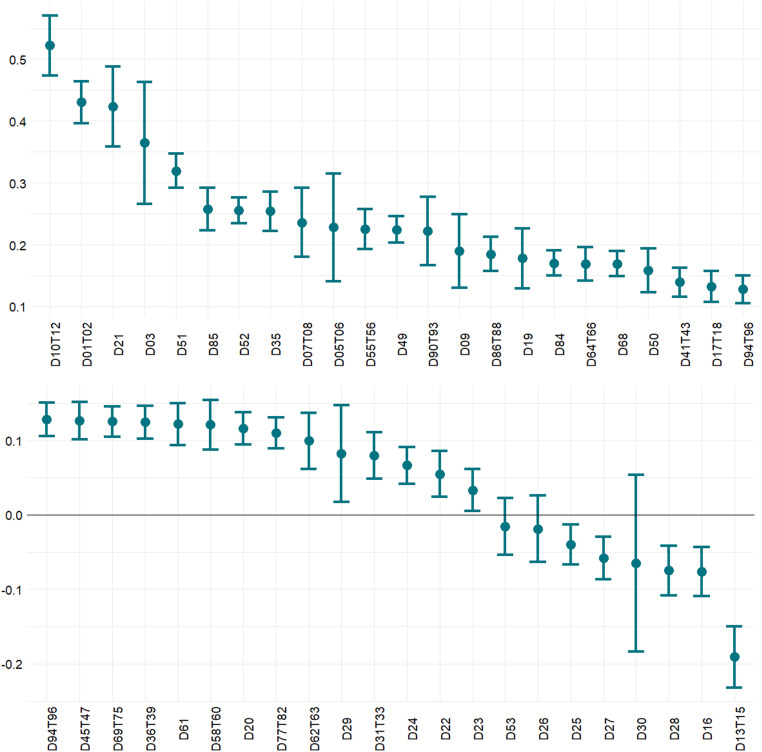
Estimated industry-specific effect of the EU enlargement. The dots refer to point estimates while the vertical lines stand for the 95% confidence intervals. The codes on the horizontal axis represent the industry according to the TiVA classification which bases on the NACE rev 2. classification. The full list of codes can be found here: https://ec.europa.eu/competition/mergers/cases/index/nace_all.html.

Fig 1 portrays the industry-specific effects of European integration. Although the documented heterogeneity is quite substantial because the estimates range from −*.*2 to*.*5 several conclusions can be drawn. Firstly, the overall effect is positive for most industries. Secondly, the largest gains are observed in the agricultural sector (D01T02) as well as the manufacturing of food, beverages, and tobacco products (D10TD12). Therefore, the large effect in those industries can be explained by a high degree of external EU protectionism before the enlargement and a substantial role of trade barriers (i.e., trade costs) that were removed with the EU enlargement. It must be mentioned that one could also expect this to result from the Common Agricultural Policy in the EU, but this policy is unilateral in nature and hence, it is captured by the time-varying fixed effects. Thirdly, services (sectors beyond D40) exhibit relatively higher gains than the manufacturing industry, which may be on one hand related to freedom of movement and residence for citizens within the EU as well as the fact that even restricted access to the service markets (which has been liberalized later in the form of the Service Directive) was substantially improved through EU enlargement.

The negative effects are reported for industries that are most spectacular examples of the activity within the global value chains, i.e., manufacturing of machinery and equipment (D28), electrical equipment (D27), other transport equipment (D30), as well as manufacturing of metal products (D25). The counterintuitive estimates here can be explained by the fact that production within these industries is extremely specialized and fragmented, not only between European economies but over many countries. The additional key feature of those industries is that they produce investment goods which production requires complementary (to investment goods) but specific technologies that are not common and developed in several countries in which the R&D centres are located. Given that the R&D activity is to a large extent concentrated in a few economies this implies that the supply chains producing investment goods could be quite complex and cross the EU border. Finally, this can also mean that as the NMS occupy the low-value-added part of the value chain, while the EU enlargement improved the trade volume, the value added per unit of output in those industries could have fallen leading to an overall drop in exported value added. In comparison, the estimated value-added premium from EU enlargement for manufacturing of motor vehicles, trailers, and semi-trailers (D29) is significantly positive because this branch offers mostly consumption goods (that are later retailed) rather than investment goods. Similar conclusions apply to pharmaceuticals (D21), a sector that additionally has relatively high R&D intensity.

## 6 Estimating the effects of Brexit on GDP

Finally, we assess the *ex-ante* effects of Brexit on GDP among exporters. Our empirical strategy has an advantage related to measurement. Namely, the estimated effect of the European integration can be translated directly into gains in value added (and hence, GDP). To scrutinize the macroeconomic effect, these estimates are combined with information about the structure of the final absorption of value added. We consider two cases: (i) a homogeneous effect, (ii) an industry-specific effect of Brexit. In the first case, the macroeconomic effect represents the estimated effect of Brexit on $VA_{ijt}$ multiplied by the share of value added produced in a given economy and finally absorbed in the UK. In the second strategy, the industry-specific effects of Brexit on the $VA_{ijt}$ are aggregated by using the shares of the industry’s value added that is absorbed in the UK. In both cases we use the structure of value added obtained with the Leontief inverse and the TiVA data in 2018.

We then re-estimate the structural gravity model with an additional variable that is the interaction between the $EU_{ijt}$ and a dummy indicating that the value added is absorbed in the United Kingdom ($IMP_{ijt}^{GBR}$). The motivation for such a re-setting is related to the efficiency of estimation. Although our previous inspection of heterogeneity has illustrated that the estimated effect of European integration could vary a lot, we should keep in mind that we are interested in the specific effects of Brexit on CEE trade with the UK. The expected size of the coefficient is not clear as the different features of the UK’s economy affect trade in opposite ways: (i) it is one of the largest importers in the EU (which increases trade) but (ii) a rather distant market geographically, in particular to the CEE.

Table 4 presents the extended estimates. In fact, the effect of EU integration on exports of value added that is ultimately absorbed in the UK final demand is about two times higher than the average effect. This premium is present irrespective of the choice of the dataset (aggregate versus sectoral data). Importantly, estimates on control variables remain very close to the previous results. This result stems from the fact, that the UK economy is relatively open both in merchandise trade and services and trades more with the rest of the EU than it is predicted by the regular gravity variables.

**Table 4:** Estimated effect of Brexit on exported value added.

	(1)	(2)
	Aggregate data	Sectoral data
$EU_{ijt}$	0.123***	0.122***
	(0.0103)	(0.00346)
$wto_{ijt}$	0.0667*	0.0538***
	(0.0364)	(0.0132)
$rta_{ijt}$	0.0240***	0.0142***
	(0.00741)	(0.00267)
$EU_{ijt}\times IMP_{ijt}^{GBR}$	0.128***	0.158***
	(0.0282)	(0.0101)
*N*	102,960	4,497,147
$R^{2}$	0.996	0.994

Note: ***, ** and * denote the rejection of null about parameters’ insignificance at 1%, 5% and 10% significance level, respectively. The expressions in round brackets stand for robust standard errors. Estimations based on the TiVA dataset for the period of 1995–2018.

**Fig 2:**
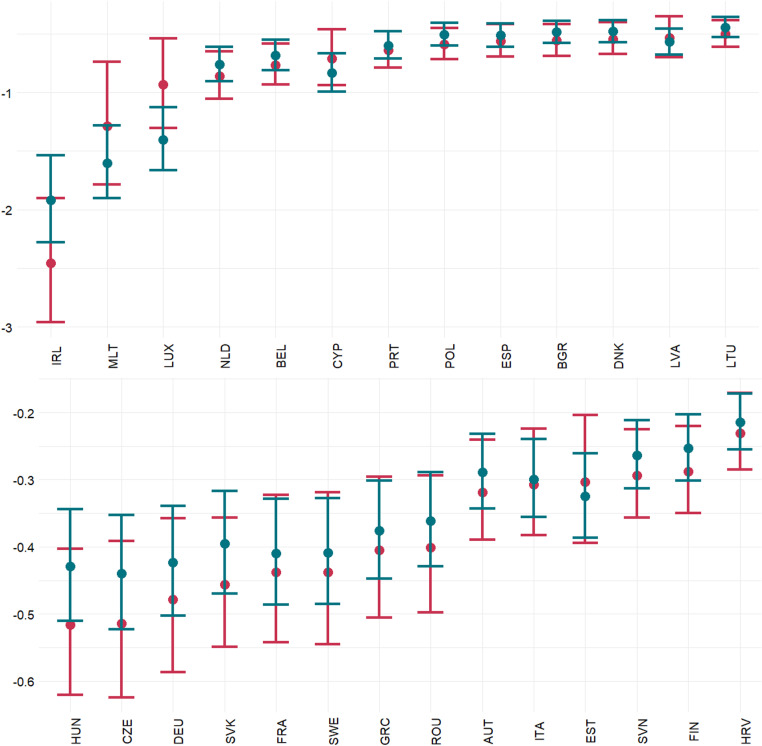
Estimated effect of Brexit (in % of GDP). The dots refer to point estimates while the vertical lines stand for the 95% confidence intervals. The green and red colours denote the estimates assuming the homogeneous and industry-specific effect of Brexit, respectively.

The estimated macroeconomic implications of Brexit are illustrated in Fig 2. To check robustness, we also provide calculations based on estimated effects from (i) the bilateral dataset (estimates in column (1) in Table 4) and (ii) the industry-specific estimations of the structural gravity model (the detailed results are available upon request but the remaining estimates, i.e., in column (2) in Table 4, provide the same picture). Clearly, the effect is the highest for economies for which the UK is the key final destination. Malta and Ireland can expect a drop of more than 3% of GDP. For the rest of exporters, potential losses in GDP do not exceed 1% of GDP (but are statistically significant). Importantly, the results are not biased by the structure of exports. This is confirmed by the similarity of estimates based on the bilateral dataset and industry-specific estimates accounting for heterogeneous industry effects.

## 7 Robustness check: WIOD dataset

TiVA is not the only database available that covers the trade in value added indicators. As the construction of the multi-country input-output tables requires numerous assumptions, the data itself can be a source of uncertainty of the obtained results. In order to check for robustness, we employ the alternative available dataset, the World Input Output Database (hence after WIOD) [36]. It offers data on 56 sectors in 43 countries over time span ranging from 1995 to. We use two editions of WIOD that span different time periods (1995–2009 for the 2013 edition and 2000–2014 for the 2016 edition). Since the sectoral classifications differ across the two datasets, we match the conflicting sectors by joining them to higher-level aggregates.

Given the different time coverage, country coverage and sectoral definitions of the alternative dataset, the results obtained in the previous section are highly robust. The estimates obtained using WIOD (see Table 5) are almost the same as the baseline estimates, in particular with regard to the effect of EU enlargement. The estimates of the effects of WTO membership and RTA membership are, however, considerably lower which is likely due to the considerably lower coverage of non-EU countries in the case of WIOD.

**Table 5:** Alternative estimated effect of European integration on exported value added.

	(1)	(2)	(3)	(4)	(5)	(6)
	Aggregate data			Sectoral data		
$EU_{ijt}$	0.130 ***	0.129 ***	0.129 ***	0.125 ***	0.124 ***	0.124 ***
	(0.0361)	(0.0359)	(0.0359)	(0.032)	(0.032)	(0.032)
$wto_{ijt}$		0.202 **	0.201 **		0.156 *	0.155 *
		(0.082)	(0.082)		(0.080)	(0.080)
$rta_{ijt}$			0.018			0.0147
			(0.022)	(0.017)		
*N*	3,489	3,489	3,489	1,811,532	1,811,532	1,811,532
$R^{2}$	0.997	0.997	0.997	0.994	0.994	0.994

Note: ***, ** and * denote the rejection of null about parameters’ insignificance at 1%, 5% and 10% significance level, respectively. The expressions in round brackets stand for robust standard errors. Estimations based on the WIOD dataset for the period of 1995–2014.

Similar conclusions apply to other estimates. While the WIOD-based versions of all the estimations are available on request from the authors, in Table 6 we show the estimates of the gravity model that focuses on the effect of EU enlargements on trade with the UK with the appropriate interaction of variables. These results are, again, of a very similar order of magnitude to those presented in Table 4, with regard to the main variables of interest.

**Table 6:** Alternative estimated effect of Brexit on exported value added.

	(1)	(2)
	Aggregate data	Sectoral data
$EU_{ijt}$	0.120 ***	0.116 ***
	(0.036)	(0.036)
$wto_{ijt}$	0.201 **	0.156 **
	(0.081)	(0.080)
$rta_{ijt}$	0.018	0.015
	(0.022)	(0.017)
$EU_{ijt}\times IMP_{ijt}^{GBR}$	0.182 ***	0.164 ***
	(0.069)	(0.059)
*N*	3,489	1,811,532
$R^{2}$	0.997	0.994

Note: ***, ** and * denote the rejection of null about parameters’ insignificance at 1%, 5% and 10% significance level, respectively. The expressions in round brackets stand for robust standard errors. Estimations based on the WIOD dataset for the period of 1995–2014.

## 8 Conclusions

In this paper, we revisit the topic of trade effects of EU enlargement. Unlike most of the existing literature, we focus on trade in value added. This makes a sizeable difference in terms of interpretation of the results; while the boost in gross trade after EU enlargement has been sizeable, some of it has been due to the process of increased production fragmentation—increasing share of foreign value added in exports and therefore a lower per output unit domestic value added with higher gross trade volume.

This is indeed confirmed by our results. The baseline estimates point to an increase in the aggregate exported value added by 13.9% with a similar effect stemming from the estimates of sectoral statistics. In analyzed studies, estimates of trade effects of EU enlargement and EU integration obtained for the gravity models range from non-significant in early studies range from non-significant [18] and about 15% [19] to considerably larger in recent studies based on structural gravity (up to 40% in [31] and 50% in [8]). The above studies all utilise gross statistics where a simultaneous boost in imports and exports is understood as a trade expansion. In our approach, if such an expansion in trade is associated with an increase in the foreign value added content of exports, the boost in value added exports is considerably smaller than that of gross trade. Evidence provided in, e.g., [11] shows that the domestic value added in exports indeed decreased, while foreign increased in the New Member States in the period around the time of EU enlargement, supporting the above conclusion.

While our results show a sizeable boost in the exports of value-added on average, there is also great heterogeneity in the effect. This heterogeneity is present in both the country and the sector dimensions. Surprisingly, in several important export-oriented manufacturing sectors, the exports of value added did not experience a significant increase or even fell. A large increase of exports of value-added has been noted in the services sectors, where exports grew either directly (in the form of gross exports) or embedded in the exports of manufactures. A large increase in exported value-added was also experienced in the agri-food trade.

Our augmented gravity results allow ex-ante predictions of the effect of Brexit, which to a large extent is a reversal of the integration process. While the UK will still have a free trade area with the EU, the agreement is a similar arrangement to the one with the CEE countries before the EU enlargements. The UK is a important trading partner for the EU - estimated trade effect of economic integration with the UK is twice the size of the average effect of European integration (in total, around 28%). Based on these estimates, we compute the likely trade effect of Brexit, stemming from the re-introduction of non-tariff barriers to trade. This effect is estimated to be less than 1% of GDP for most of the EU members (except Malta and Ireland, where it is significantly larger). It has to be noted, that this is a static effect, i.e., it does not take into account the long-term effects of capital (dis)accumulation. Comparing these results with those found in the literature shows that our estimates are in the ballpark of those found in the literature. For example, the short-run trade effects obtained in [26] for all the EU countries amount to 13–38% of lost trade depending on a scenario. The country-level simulated aggregate real consumption pattern shown in [29] shows a similar country spread as in our results for the macroeconomic effect of Brexit in terms of GDP – with the highest effects of the EU members in Ireland (3.1%) and the lowest in Croatia (0.04%) in the FTA scenario similar to the actual outcome of Brexit. The existing literature offers a wide range of estimates of macroeconomics effects Brexit. For example, the welfare changes shown in [30] are in general lower than our estimates and lower than other studies found in the literature.

It has to be noted that the estimated effects of Brexit are still ex-ante effects. The full ex-post evaluation of trade and macroeconomic effects will be possible when sufficient trade data is available. Once they are, our approach to the gravity model estimation based on value added flows can also be used for direct estimation of the trade effects of Brexit and these results can be directly translated into trade-induced changes in GDP isolated from other likely effects of Brexit that are unrelated to trade (i.e., participation in EU programmes and budget, reputation effects, etc.) as these are captured in our framework by time-varying fixed effects.

Our paper offers important insights into the policy implications of European integration: EU enlargement and Brexit. While we show that while the impact of the removal of trade barriers through European integration on the level of value added trade in almost all analysed cases is positive, it is by no means uniform. There is a great deal of country-specific and sector-specific heterogeneity. This applies not only to the effects of enlargement but also to the estimated effects of Brexit. Looking beyond Brexit, these results show that the support for integration may vary among the EU countries and depend on their sectoral composition.

## Supporting information

S1 Table Definitions of variables used in estimations. (DOCX)

S2 Table Descriptive statistics of variables. (DOCX)

S3 Table Pairwise correlation of variables. (DOCX)

S1 Fig Heterogeneous export- and import-specific estimated effects of EU enlargements. The dots refer to point estimates while the vertical lines stand for the 95% confidence intervals. The green and red colours denote the estimates for exporters and importers, respectively.
(TIF)
