# Supplementary material for: EU enlargements, Brexit and value-added trade: A structural gravity approach
Source: PLoS One. 2025 Apr 16;20(4):e0299738. doi: 10.1371/journal.pone.0299738 (PMC12002806; doi:10.1371/journal.pone.0299738)
Supplement: S1 Table — (DOCX) [file pone.0299738.s001.docx]

| Variable | Description | Source |
| --- | --- | --- |
| $VA_{ijt}$ | Domestic value added exporter by country *i* and absorbed in final demand in country *j* at time *t* (millions of USD) | OECD TiVA database |
| $VA_{ijst}$ | Domestic value added exported by sector *s* country *i* and absorbed in final demand in country *j* at time *t* (millions of USD) | OECD TiVA database |
| $\mathcal{E}\mathcal{U}_{ijt}$ | A dummy variable taking the value of 1 if both country *i* and *j* at time *t* are members of the EU and zero otherwise | CEPII gravity database |
| $wto_{ijt}$ | A dummy variable taking the value of 1 if both country *i* and *j* at time *t* are members of the WTO and zero otherwise | CEPII gravity database |
| $rta_{ijt}$ | A dummy variable taking the value of 1 if both country *i* and *j* at time *t* are members of the regional preferential trading agreement and zero otherwise | CEPII gravity database |
| $logdist_{ij}$ | Logarithm of distance between main cities in country *i* and *j* (log kilometers*)* | CEPII gravity database |
| $log{GDP}_{it}$ | Logarithm of gross domestic product of exporter *i* (log millions of USD) | CEPII gravity database |
| $log{GDP}_{jt}$ | Logarithm of gross domestic product of importer *j* (log millions of USD) | CEPII gravity database |
| $IMP_{ijt}^{GBR}$ | A dummy variable that takes a value of 1 if the importer is the United Kingdom and zero otherwise | Based on country names |
| $\mathcal{E}\mathcal{U}_{ijt}\times logdist_{ij}$ | Interaction between $\mathcal{E}\mathcal{U}_{ijt}$ and $logdist_{ij}$ variable | Based on existing variables |
| $\mathcal{E}\mathcal{U}_{ijt}\times log{GDP}_{it}$ | Interaction between $\mathcal{E}\mathcal{U}_{ijt}$ and $log{GDP}_{it}$variable | Based on existing variables |
| $\mathcal{E}\mathcal{U}_{ijt}\times log{GDP}_{jt}$ | Interaction between $\mathcal{E}\mathcal{U}_{ijt}$ and $log{GDP}_{jt}$variable | Based on existing variables |
| $\mathcal{E}\mathcal{U}_{ijt}\times IMP_{ijt}^{GBR}$ | Interaction between $\mathcal{E}\mathcal{U}_{ijt}$ and  $IMP_{ijt}^{GBR}$variable | Based on existing variables |
