# Supplementary material for: EU enlargements, Brexit and value-added trade: A structural gravity approach
Source: PLoS One. 2025 Apr 16;20(4):e0299738. doi: 10.1371/journal.pone.0299738 (PMC12002806; doi:10.1371/journal.pone.0299738)
Supplement: S2 Table — (DOCX) [file pone.0299738.s002.docx]

| Variable | Mean | St. Dev. | Min. | Max. |
| --- | --- | --- | --- | --- |
| $logVA_{ijt}$ | 5.04 | 2.496 | -3.817 | 13.038 |
| $log(VA_{ijst}+1)$ | 1.489 | 1.674 | 0 | 11.096 |
| $\mathcal{E}\mathcal{U}_{ijt}$ | 0.121 | 0.326 | 0 | 1 |
| $rta_{ijt}$ | 0.36 | 0.48 | 0 | 1 |
| $wto_{ijt}$ | 0.858 | 0.349 | 0 | 1 |
| $log{GDP}_{it}$ | 19.009 | 1.785 | 14.063 | 23.748 |
| $log{GDP}_{jt}$ | 19.009 | 1.785 | 14.063 | 23.748 |
| $logdist_{ij}$ | 8.501 | 1.007 | 4.088 | 9.894 |
| $\mathcal{E}\mathcal{U}_{ijt}\times log{GDP}_{it}$ | 2.326 | 6.293 | 0 | 22.1 |
| $\mathcal{E}\mathcal{U}_{ijt}\times log{GDP}_{jt}$ | 2.326 | 6.293 | 0 | 22.1 |
| $\mathcal{E}\mathcal{U}_{ijt}\times logdist_{ij}$ | 0.854 | 2.316 | 0 | 8.234 |
| $\mathcal{E}\mathcal{U}_{ijt}\times IMP_{ijt}^{GBR}$ | 0.005 | 0.071 | 0 | 1 |
