# Supplementary material for: EU enlargements, Brexit and value-added trade: A structural gravity approach
Source: PLoS One. 2025 Apr 16;20(4):e0299738. doi: 10.1371/journal.pone.0299738 (PMC12002806; doi:10.1371/journal.pone.0299738)
Supplement: S3 Table — (DOCX) [file pone.0299738.s003.docx]

| Variable | $logVA_{ijt}$ | $\mathcal{E}\mathcal{U}_{ijt}$ | $rta_{ijt}$ | $wto_{ijt}$ | $log{GDP}_{it}$ | $log{GDP}_{jt}$ | $logdist_{ij}$ | $\mathcal{E}\mathcal{U}_{ijt}\times log{GDP}_{jt}$ | $\mathcal{E}\mathcal{U}_{ijt}\times log{GDP}_{jt}$ | $\mathcal{E}\mathcal{U}_{ijt}\times logdist_{ij}$ | $\mathcal{E}\mathcal{U}_{ijt}\times IMP_{ijt}^{GBR}$ |  |
| --- | --- | --- | --- | --- | --- | --- | --- | --- | --- | --- | --- | --- |
| Aggregate data | | | | | | | | | | | | |
| $logVA_{ijt}$ | 1 |  |  |  |  |  |  |  |  |  |  |  |
| $\mathcal{E}\mathcal{U}_{ijt}$ | 0.198 | 1 |  |  |  |  |  |  |  |  |  |  |
| $rta_{ijt}$ | 0.198 | 0.495 | 1 |  |  |  |  |  |  |  |  |  |
| $wto_{ijt}$ | 0.22 | 0.151 | 0.225 | 1 |  |  |  |  |  |  |  |  |
| $log{GDP}_{it}$ | 0.668 | 0.045 | 0.019 | 0.198 | 1 |  |  |  |  |  |  |  |
| $log{GDP}_{jt}$ | 0.635 | 0.045 | 0.019 | 0.198 | 0.052 | 1 |  |  |  |  |  |  |
| $logdist_{ij}$ | -0.227 | -0.527 | -0.623 | -0.019 | 0.054 | 0.054 | 1 |  |  |  |  |  |
| $\mathcal{E}\mathcal{U}_{ijt}\times log{GDP}_{it}$ | 0.213 | 0.996 | 0.492 | 0.15 | 0.071 | 0.043 | -0.527 | 1 |  |  |  |  |
| $\mathcal{E}\mathcal{U}_{ijt}\times log{GDP}_{jt}$ | 0.212 | 0.996 | 0.492 | 0.15 | 0.043 | 0.071 | -0.527 | 0.992 | 1 |  |  |  |
| $\mathcal{E}\mathcal{U}_{ijt}\times logdist_{ij}$ | 0.186 | 0.995 | 0.492 | 0.15 | 0.041 | 0.041 | -0.501 | 0.99 | 0.99 | 1 |  |  |
| $\mathcal{E}\mathcal{U}_{ijt}\times IMP_{ijt}^{GBR}$ | 0.096 | 0.191 | 0.095 | 0.029 | 0.006 | 0.102 | -0.107 | 0.19 | 0.218 | 0.188 | 1 |  |
| Sectoral Data | | | | | | | | | | | | |
| $log(VA_{ijst}+1)$ | 1 | 0.178 | 0.152 | 0.147 | 0.521 | 0.484 | -0.195 | 0.195 | 0.194 | 0.165 | 0.101 |  |
