# Supplementary figures and images for: EU enlargements, Brexit and value-added trade: A structural gravity approach
Source: PLoS One. 2025 Apr 16;20(4):e0299738. doi: 10.1371/journal.pone.0299738 (PMC12002806; doi:10.1371/journal.pone.0299738)

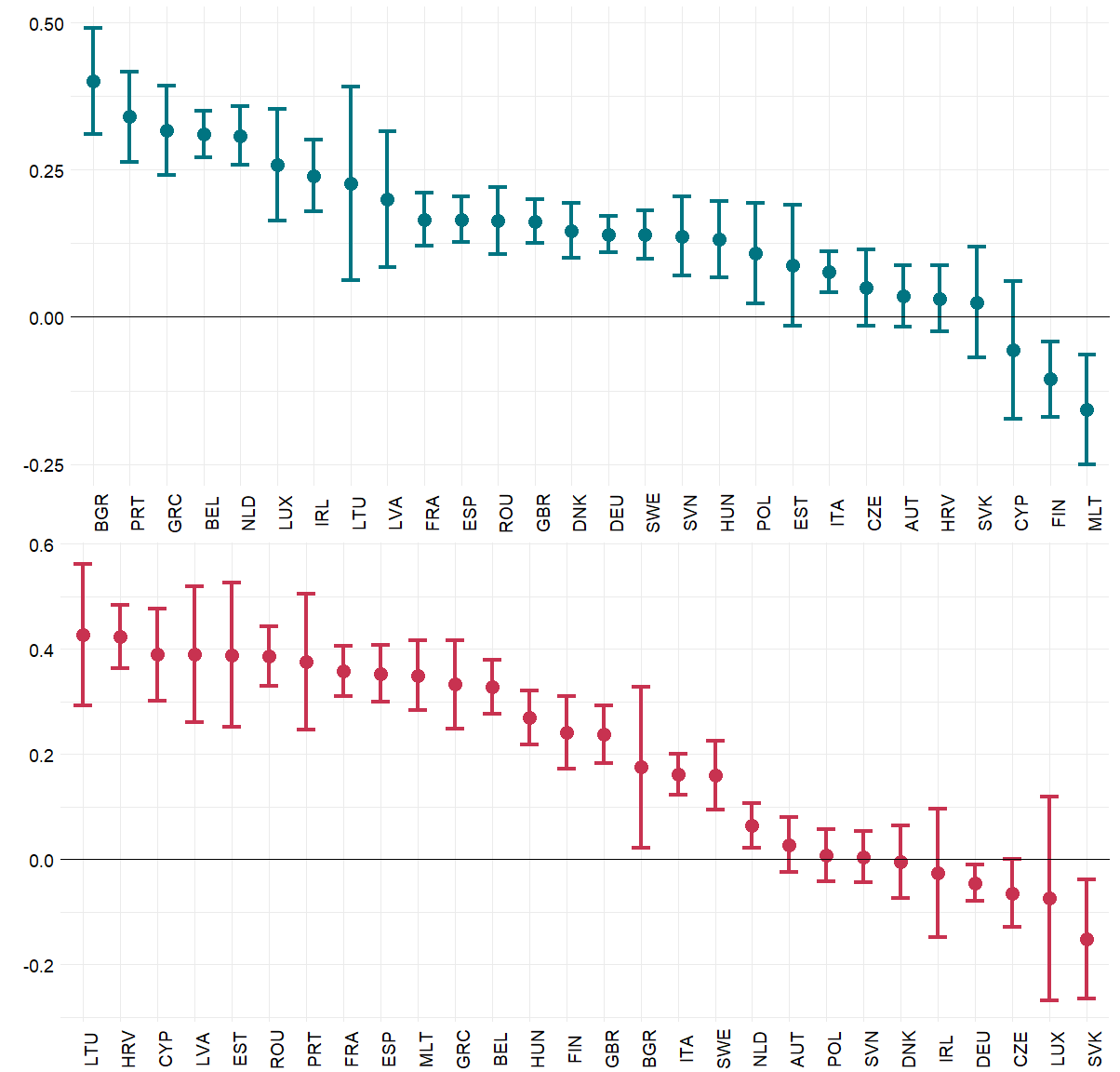

Supplement: S1 Fig — The dots refer to point estimates while the vertical lines stand for the 95% confidence intervals. The green and red colours denote the estimates for exporters and importers, respectively. (TIF) [file pone.0299738.s004.tif]
